# Diabetes e Insuficiencia Cardíaca

**DOI:** 10.47487/apcyccv.v1i1.5

**Published:** 2020-03-30

**Authors:** Walter Alarco

**Affiliations:** 1 Unidad de Insuficiencia Cardiaca, Trasplante Cardiaco e Hipertensión Pulmonar Instituto Nacional Cardiovascular INCOR. Lima, Perú.

**Keywords:** diabetes, insuficiencia cardíaca, diabetes, heart failure

## Abstract

La Diabetes Mellitus (DM) es una enfermedad crónica cardiometabólica no transmisible, que origina complicaciones macrovasculares como aterosclerosis, enfermedad coronaria e insuficiencia cardíaca (IC). Existe una relación bidireccional entre IC y DM, siendo IC el segundo evento cardiovascular inicial más frecuente en pacientes con diabetes. Incluso puede ser la primera complicación cardiovascular, antes que el infarto de miocardio.

La DM puede conducir a IC a través de mecanismos mediados por aterosclerosis e independientemente de esta. En el primer caso, se produce depósito de colesterol en las arterias coronarias, favorecido por la presencia de otros factores de riesgo. En el segundo caso, a través de mecanismos no aterogénicos (denominada Cardiomiopatía Diabética), conduciendo a cambios estructurales y funcionales.

El tratamiento de la IC con fracción de eyección reducida en el paciente diabético no difiere del de la población no diabética. Se debe conseguir el triple bloqueo neurohumoral. En el caso de los pacientes con IC con fracción de eyección preservada hasta la fecha no tenemos terapia específica que disminuya la morbimortalidad cardiovascular.

En el tratamiento de la diabetes del paciente con IC sobresalen claramente los inhibidores del cotransportador sodio-glucosa tipo 2 (iSGLT2) que además de su efecto glucosúrico y natriurético, presentan efectos pleiotrópicos que ejercen una acción metabólica, hemodinámica y en la viabilidad celular al prevenir apoptosis y muerte celular. Finalmente, los beneficios clínicos de los inhibidores SGLT2 en IC van mas allá del control glicémico, como lo demostró el estudio DAPA-HF; iniciando una nueva era en el tratamiento del paciente con IC con fracción de eyección reducida.

## Abstract (en)

Diabetes Mellitus (DM) is a chronic non-communicable cardio-metabolic disease that causes macro-vascular complications such as atherosclerosis, coronary disease and heart failure (HF). There is a bidirectional relationship between HF and DM, HF being the second most frequent initial cardiovascular event in patients with diabetes. It may even be the first cardiovascular complication, before acute myocardial infarction.

DM can lead to HF through mechanisms mediated by atherosclerosis and non mediated by it. In the first case, cholesterol is deposited in coronary arteries, favored by the presence of other risk factors. In the second case, the myocardium is directly affected leading to structural and functional changes through non-atherogenic mechanisms (called Diabetic Cardiomyopathy).

The treatment of HF with reduced ejection fraction in the diabetic patient does not differ from that of the non-diabetic population, the triple neurohumoral block must be achieved. In the case of patients with HF with ejection fraction preserved to date, we do not have specific therapy to reduce cardiovascular morbidity and mortality.

In the diabetes treatment of the patient with HF, sodium-glucose type 2 cotransporter inhibitors (SGLT2i) clearly stand out. In addition to their glucosuric and natriuretic effect, they have pleiotropic effects that produce metabolic, hemodynamic and cellular viability effects preventing apoptosis and cell death. Finally, the clinical benefits of SGLT2 inhibitors in HF go beyond glycemic control, as demonstrated by the DAPA-HF study; initiating a new era in the treatment of patients with HF with reduced ejection fraction.

La diabetes mellitus (DM) es una de las enfermedades crónicas no transmisibles que más ha crecido en los últimos años, tanto por su prevalencia y creciente incidencia; así como por su gran relevancia al tratarse de un factor de riesgo independiente para desarrollar enfermedad cardiovascular aterosclerótica e insuficiencia cardiaca (IC). 

La causa primaria de mortalidad en pacientes con diabetes es la enfermedad cardiovascular, que corresponde al 50-80% de las muertes. ^(^^1^ La American Diabetes association (ADA) estima que el 50% de los pacientes con DM tendrán IC a lo largo de su vida. ^2^

En el siguiente artículo trataremos de revisar la magnitud del problema, dar una explicación a los mecanismos implicados en la cardiomiopatía diabética y sobretodo, hacer un resumen de los nuevos tratamientos farmacológicos para la diabetes con énfasis en los inhibidores del cotransportador de sodio-glucosa tipo 2 (iSGLT2) y su impacto en la prevención primaria de insuficiencia cardiaca (nuevos casos) y el tratamiento de los pacientes con enfermedad establecida.

## Magnitud del Problema

La DM es una enfermedad cardio metabólica que origina complicaciones microvasculares y macrovasculares, las cuales afectan a múltiples órganos, y cuya prevalencia viene en aumento. ^3^ Aproximadamente el 10% de la población mundial padece de DM tipo 2 y el 2-3% de insuficiencia cardiaca. Se ha establecido que existe una relación bidireccional entre ambas patologías; múltiples causas comunes llevan a vías comunes fisiopatológicas que resultan en un efecto deletéreo de la DM sobre la IC. La consecuencia clínica es que el paciente con ambas enfermedades presenta peor clínica y peor pronóstico que el paciente con IC sin DM, independientemente de la fracción de eyección. ^4^

La prevalencia de DM a través de pacientes con IC crónica es 25% y en IC aguda es 40-45%, mientras la prevalencia de IC es 10-23% en los pacientes diabéticos. Los hombres diabéticos tienen 2.4 veces mayor probabilidad de desarrollar insuficiencia cardiaca y las mujeres el riesgo se multiplica por 5, desarrollando éstas últimas, insuficiencia cardiaca con fracción de eyección preservada (ICFEp) predominantemente. ^5^ La IC también incrementa el riesgo de incidencia de DM tipo 2. En Estudios como CHARM^6^ y EMPHASIS-HF^7^ se reportó incremento de incidencia de DM (28 y 21 por 1000 personas-año, respectivamente).

En pacientes con IC sin DM, 20-43% tienen intolerancia a la glucosa y se traduce en mayor mortalidad^8^ además, en los pacientes con DM, por cada aumento del 1% en la hemoglobina glicosilada, el riesgo de IC se incrementa en 8 %. ^9^ Sin embargo, el desarrollo de IC en diabéticos está más estrechamente relacionado con la duración de la DM. Aunque varios estudios han demostrado el beneficio del óptimo control glicémico sobre las complicaciones microvasculares, aún queda poco claro los efectos del control estricto glicémico sobre las complicaciones macrovasculares; los últimos ensayos clínicos expresan que la prevención y tratamiento de las complicaciones cardiovasculares, como la insuficiencia va más allá del control glicémico. 

La IC es el segundo evento cardiovascular (CV) inicial más frecuente en pacientes con DM, incluso tiene mayor probabilidad de ser la primera complicación CV, antes que el infarto agudo al miocardio o el accidente cerebrovascular. ^10^

Lo cierto es que ambas patologías son un problema de salud pública en crecimiento. El impacto en cuanto a mortalidad y hospitalizaciones por insuficiencia cardiaca es preocupante, más aún de lo segundo; lo que repercute en el mal pronóstico de los pacientes, alta tasa de readmisión y altos costos directos e indirectos al sistema de salud. El paciente con IC y DM tiene 33% mayor riesgo de hospitalización que un paciente no diabético. ^11^

## Cardiomiopatía Diabética

La DM puede conducir a IC a través de mecanismos mediados por aterosclerosis e independientemente de aterosclerosis. Por un lado, la DM per se y la combinación con otros factores de riesgo cardiovascular como la hipertensión arterial y dislipidemia; favorecen la disfunción endotelial, la inflamación, stress oxidativo y el daño vascular, lo que se traduce en enfermedad coronaria, isquemia miocárdica y miocardiopatía isquémica. Asimismo, existen mecanismo no aterogénicos, por los cuales la DM afecta directamente el miocardio conduciendo a cambios estructurales y funcionales a nivel del corazón. Entre los mecanismos implicados podemos mencionar: la glucotoxicidad directa producida por la hiperglicemia/resistencia insulina, activación del sistema-renina-angiotensina-aldosterona, stress oxidativo, alteración SERCA-2, disfunción autonómica, microangiopatía, disfunción mitocondrial, lipotoxicidad y esteatosis miocárdica, necrosis miocárdica subclínica, apoptosis, anormalidades en la bomba Na/H y alteración en la reserva energética del cardiomiocito. ^(^^12^^-^^19^

**Figura 1:**
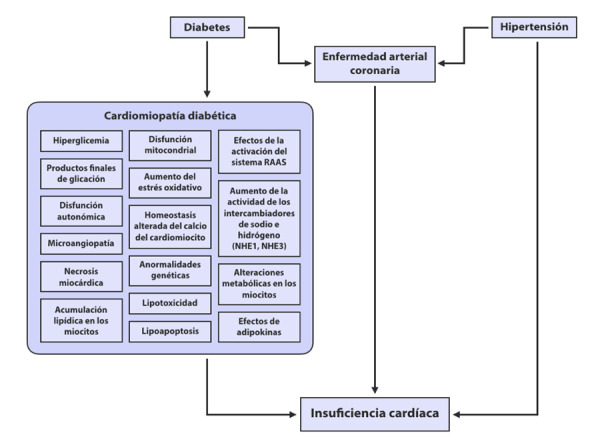
Cardiomiopatía Diabética. Adaptado de: Wilkinson MJ. Am J Cardiol 2019;124: S3-S11

Todos estos mecanismos contribuyen en alguna medida en el incremento de la fibrosis intersticial, remodelado e hipertrofia ventricular. (Figura 1)

Las alteraciones funcionales, ocurren principalmente a nivel mitocondrial de los miocitos y traen como consecuencia una alteración de la contractilidad miocárdica. El efecto de la hiperglicemia crónica y la resistencia a la insulina afecta el manejo del calcio intracelular. La recaptación de calcio y su sobrecarga a nivel intracelular altera la diástole y en muchos casos, progresa a disfunción sistólica.

El diagnóstico de cardiomiopatía diabética requiere una historia de varios años de diabetes, pobre control glicémico y descartar otras etiologías de la IC. Se caracteriza por disfunción diastólica temprana en ausencia de enfermedad coronaria o enfermedad cardiaca hipertensiva. Sin embargo, deberíamos resaltar que este tipo de cardiomiopatía puede estar presente simultáneamente con otras condiciones cardiovasculares; pero será la diabetes per se, la que explique el daño miocárdico inicial. 

Se identifican dos fenotipos: la cardiomiopatía dilatada y el fenotipo restrictivo, en el cual predomina el remodelado concéntrico y la disfunción diastólica, en su evolución natural desarrollan IC con fracción de eyección reducida (ICFEr) o insuficiencia cardiaca con fracción de eyección preservada (ICFEp) respectivamente. ^(^^20^^-^^23^

Los estudios de imágenes y los biomarcadores séricos son de suma utilidad en el diagnóstico de la enfermedad clínica y subclínica. La disfunción diastólica es un hallazgo frecuente en los pacientes con DM y tiene una incidencia entre el 23 y 75%. En el subestudio ADVANCE la existencia de disfunción diastólica, definida como alteración de la relación E/e´ más dilatación auricular, se asoció con eventos cardiovasculares en el 33% de los pacientes. ^(^^24^

El ecocardiograma es el método más accesible para valorar la contractilidad miocárdica, fracción de eyección, hipertrofia y cuantificación de la masa ventricular, así como la función diastólica. En ese sentido, la valoración de la función cardiaca por ecocardiografía debería ser parte de la evaluación integral de los pacientes diabéticos.

El daño subclínico puede ser detectado por la Resonancia Cardiaca que es el Gold standard para la valoración de fibrosis, disfunción microvascular; y por los nuevos métodos ecocardiográficos de strain y speckle tracking. En ese sentido, la medición del strain longitudinal global (GLS) es el parámetro de deformación más utilizado en la práctica clínica.^24^^-^^27^

En relación con los biomarcadores, el proBNP es el de mayor utilidad clínica; le sigue galactina-3. La detección de la disfunción ventricular subclínica por niveles elevados de péptidos natriuréticos en el estudio PONTIAC en pacientes diabéticos de alto riesgo, demostró disminuir muerte y hospitalizaciones por causa cardiovascular, luego de un inicio precoz de terapias destinadas al tratamiento farmacológico de la IC.^28^^-^^29^

La Guía ACC/AHA 2017 recomienda con un nivel de evidencia IIA el screening con péptidos natriuréticos en aquellos pacientes en riesgo de desarrollar insuficiencia cardiaca para prevenir el desarrollo de disfunción ventricular izquierda (sistólica o diastólica) o IC de novo.^30^

## Manejo de la Insuficiencia Cardíaca en el Diabético

### Insuficiencia Cardiaca con fracción de eyección reducida

Los pacientes con IC y DM presentan tasas de mortalidad más altas. En el estudio SOLVD, los pacientes diabéticos tenían una probabilidad mayor de ser admitidos por IC (cociente riesgo 1.6) y tenían tasas más altas al año de mortalidad por todas las causas (32% vs 22%), mortalidad cardiovascular (28% vs 19%) y mortalidad relacionado con falla de bomba (11% vs 6%).^31^ Sin embargo, la misma población diabética es heterogénea y el enfoque de riesgo dependerá de factores tales como edad, tiempo de DM, comorbilidades, asociación con enfermedad coronaria, etc.

En líneas generales, las recomendaciones no difieren de los pacientes no diabéticos. El triple bloqueo neuro humoral es la piedra angular del tratamiento. Las Guías de práctica clínica recomiendan fuertemente un inhibidor de la enzima convertidora de angiotensina (ECA) o antagonista del receptor de la angiotensina (ARAII) en caso de intolerancia a los primeros, asociados a beta bloqueadores como primera línea, a ellos debería sumarse en caso de persistir sintomáticos un antagonista del receptor mineralocorticoide y sacubitril/valsartán en reemplazo del IECA/ARAII.^32^^-^^35^ Algunos puntos para destacar:

- La eficacia de los IECA/ARAII es igual en población diabética. Sin embargo, debe tenerse en cuenta en su titulación la función renal y el riesgo de hiperkalemia, por ser una población más vulnerable.

- No existen diferencias entre los beta bloqueadores aprobados en IC, los cuatro son igual de eficaces y se debe intentar dar la máxima dosis que tolere el paciente.

- La eplerenona en los estudios EMPHASIS-HF y EPHESUS^36^ ha demostrado disminuir los casos de diabetes de Novo. 

- Sacubitril/Valsartan en el estudio PARADIGM-HF^37^ demostró una disminución de los valores de hemoglobina glicosilada comparado con enalapril, lo que sugeriría una acción metabólica y de atenuación de la resistencia a la insulina. Además, demostró una disminución en el deterioro de la función renal.

- En relación con los dispositivos implantables las recomendaciones no cambian. El beneficio de la terapia de resincronización abarca a los pacientes diabéticos. No obstante, la tasa de infecciones en el sitio de implante es mayor y requiere supervisión permanente.

### Insuficiencia Cardiaca con fracción de eyección preservada 

No existe a la fecha tratamiento específico para esta condición que disminuya la morbilidad y mortalidad. El tratamiento que se brinda está dirigido a aliviar la congestión con diuréticos y tratar las comorbilidades. Quizá los ARAII, como candesartan, podría tener un efecto diferencial en disminuir las hospitalizaciones, como lo demostró el estudio CHARM.^38^ Sacubitril/valsartan en el estudio PARAGON-HF^39^ no demostró mayor beneficio en la población global, ni en el subgrupo de pacientes diabéticos. Los inhibidores iSGLT2 abren una luz de esperanza en esta población y habrá que estar atentos a los resultados de algunos ensayos clínicos en curso.

## Manejo de la Diabetes en la Insuficiencia Cardíaca

El tratamiento se inicia con las medidas de cambio de estilo de vida, una dieta saludable baja en calorías y actividad física regular según la capacidad de cada paciente. El objetivo general es alcanzar niveles de glucosa en sangre tan bajos como sea posible sin aumentar el riesgo de hipoglicemia, para ello existen una serie de fármacos que pasaremos a revisar posteriormente. Lo cierto es que un control intensivo puede también aumentar el riesgo cardiovascular y nuestra meta de hemoglobina glicosilada (HbA1C) va a depender de la antigüedad de la DM, las comorbilidades, la severidad de la IC, la expectativa de vida. Si bien idealmente, se recomienda una HbA1c menor de 7, en IC y DM valores entre 7-8% sería lo más aceptable.^40^

Haremos un resumen de los fármacos hipoglicemiantes y su beneficio en pacientes con DM e IC^41^^-^^45^

*- Metformina:* Estudios observacionales han demostrado reducir morbilidad y mortalidad. Es bien tolerado, seguro, bajo riesgo de hipoglicemia y es barato. Es recomendado como tratamiento de primera línea en muchas guías de práctica clínica.

*- Sulfonilúreas:* Mejoran el control glicémico y no generan retención de sodio. No existen estudios clínicos que hayan evaluado su seguridad cardiovascular, específicamente en IC.

*- Tiazolidinadionas:* Incrementan el riesgo de hospitalización por IC y muerte. NO están recomendados en IC.

*- Insulina:* Tiene efecto anti-natriurético dosis dependiente, y se observa ligera retención de fluidos con su uso. Es incierto si aumenta el riesgo de IC o la mortalidad.

*- Inhibidores DPP4:* Más allá del control glicémico, ninguno ha demostrado un beneficio cardiovascular. No se recomienda saxagliptina y alogliptina porque incrementa la tasa de hospitalización por IC.

*- Agonistas GLP-1:* Liraglutide en el estudio LEADER^46^ y semaglutide en el estudio SUSTAIN-6^47^ demostraron una reducción en el punto primario combinado muerte CV, IM no fatal y ACV no fatal; es decir, prevención de eventos aterotrombóticos. Sin embargo, no demostraron beneficio estadísticamente significativo en reducción de hospitalizaciones por IC.

**Figura 2:**
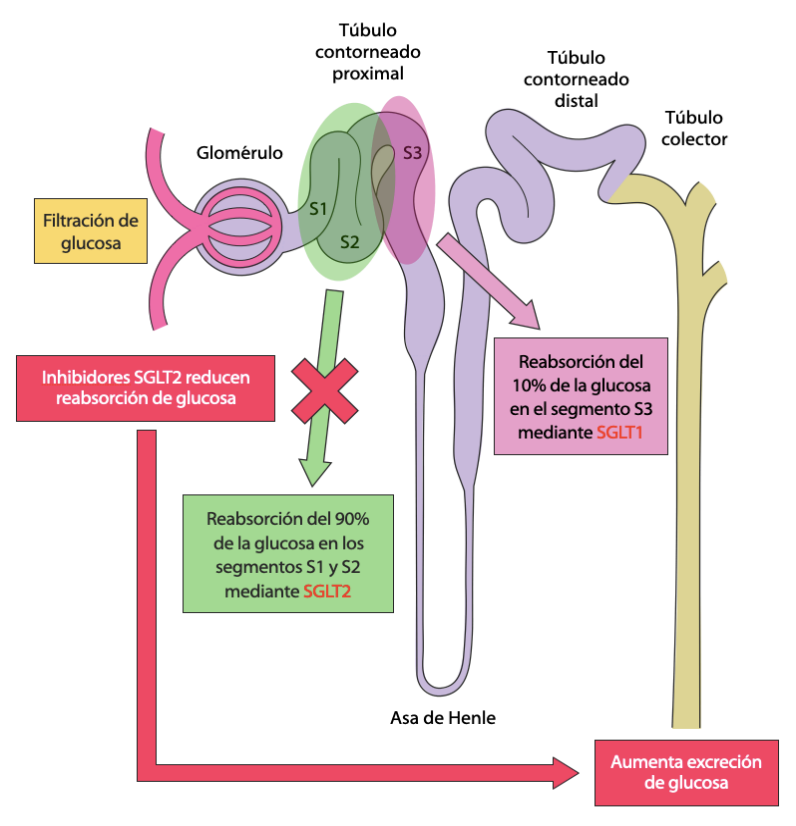
Inhibición SGLT2. Adaptado de: Perel C. Insuf Card 2018;13(4):155-169.

*- Inhibidores SGLT2:* actúan inhibiendo el cotransportador sodio-glucosa tipo 2, responsable de la mayor parte de la reabsorción de la glucosa a nivel del túbulo contorneado proximal, produciendo glucosuria y disminución de la glicemia. (Figura 2) Tanto empaglifozina (Estudio EMPA-REG^48^), canaglifozina (Estudio CANVAS^49^) y dapaglifozina (Estudio DECLARE-TIMI 58^50^), han demostrado reducir las hospitalizaciones por insuficiencia cardiaca en pacientes con y sin antecedente previo de IC. Un metaanálisis corrobora estos resultados.^51^ En la Tabla 1 se resumen las principales características de cada uno de estos estudios.

**Tabla 1:** Estudios Pivotales con los Inhibidores SGLT2: Principales Características

	EMPA-REG OUTCOME (Empaglifozina)	CANVAS (Canaglifozina)	DECLARE-TIMI 58 (Dapaglifozina)
N° pacientes	7,020	10,142	17,160
Año Publicación	2015	2017	2019
Criterio Inclusión	ECV, HBA1C 7-9%, TFG>30	ECV ó ≥50 años + ≥2 FRCV HBA1C 7-10.5%, TFG>30	ECV ó ≥50/60 (hombre/mujer) años + ≥1 FRCV, HBA1C 6.5-12%, TFG>60
Dosis	10-25 mg	100 mg	10 mg
Prevención Primaria	0 %	34.4%	59.4%
Prevención Secundaria	100 %	65.6%	40.6%
Población con insuficiencia cardíaca	10 %	14.4%	10%
Objetivo Primario	3-p MACE	3-p MACE	Co-Primario: 3p-MACE y Muerte CV / Hospitalización IC
Seguimiento	3.1 años	2.4 años	4.2 años
Resultados	HR 0.86 (0.74-0.99) p= 0.04 superioridad	HR 0.86 p=0.02 superioridad	HR 0.93 (0.84-1.03) HR 0.83 (0.73-0.95)
Reducción Hospitalización por insuficiencia cardíaca	35 %	33 %	27%

ECV: Enfermedad cardiovascular; FRCV: factores de riesgo cardiovascular; TFG: tasa filtración glomerular; 3-p MACE: Muerte cardiovascular + Infarto Miocardio no fatal + stroke no fatal; HR: hazard ratio

Si bien, no se especificaron las poblaciones con IC según su fracción de eyección en los tres estudios, datos del DECLARE demostrarían un mayor beneficio en población con ICFEr. Esta afirmación quedaría confirmada con los resultados del estudio DAPA-HF.

**Figura 3:**
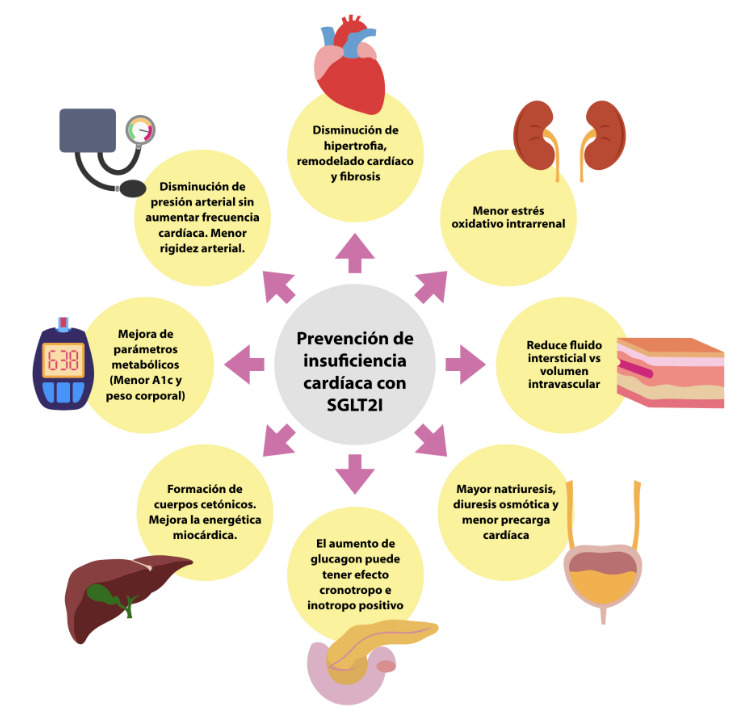
Potenciales mecanismos de los ISGLT2 en la prevención y tratamiento de la IC. Adaptado de: Butler J et al. European Journal of Heart Failure. 2020. A1C: Hemoglobina glicosilada

Lo que debemos enfatizar es que el beneficio obtenido en relación con la IC se trataría de un efecto de clase y sin duda, este grupo farmacológico tiene una clara indicación en el tratamiento de la DM en IC, tanto en la prevención de IC como en pacientes sintomáticos (Estadios A-C). Adicionalmente, es el único grupo farmacológico entre los hipoglicemiantes, que tiene un claro efecto de nefro protección y enlentecimiento en el deterioro de la función renal. ¿Qué mecanismos están involucrados y explicarían su beneficio en IC? Además de su efecto glucosúrico y natriurético, se han planteado otros efectos pleiotrópicos que en suma ejercen una acción metabólica, hemodinámica y quizá, en última instancia y no menos importante, una protección y viabilidad celular al prevenir apoptosis y muerte celular.^52^^-^^57^ (Figura 3)

## ISGLT2 en Insuficiencia Cardíaca con y sin Diabetes

La información obtenida de los ensayos clínicos mencionados previamente, así como, los buenos resultados de estudios observacionales, ha planteado la interrogante sobre si estos fármacos pueden tener un impacto positivo en el tratamiento de la IC, independientemente a que el paciente padezca de DM. En ese sentido, los resultados del estudio DAPA-HF^58^ con dapaglifozina en pacientes con ICFEr nos han abierto la posibilidad de contar con un nuevo fármaco que reduce la morbilidad y mortalidad en IC. Incluso la última Guía de Práctica Clínica de la Sociedad Canadiense de Cardiología^59^^)^ ya la incorpora entre sus recomendaciones.

**Figura 4:**
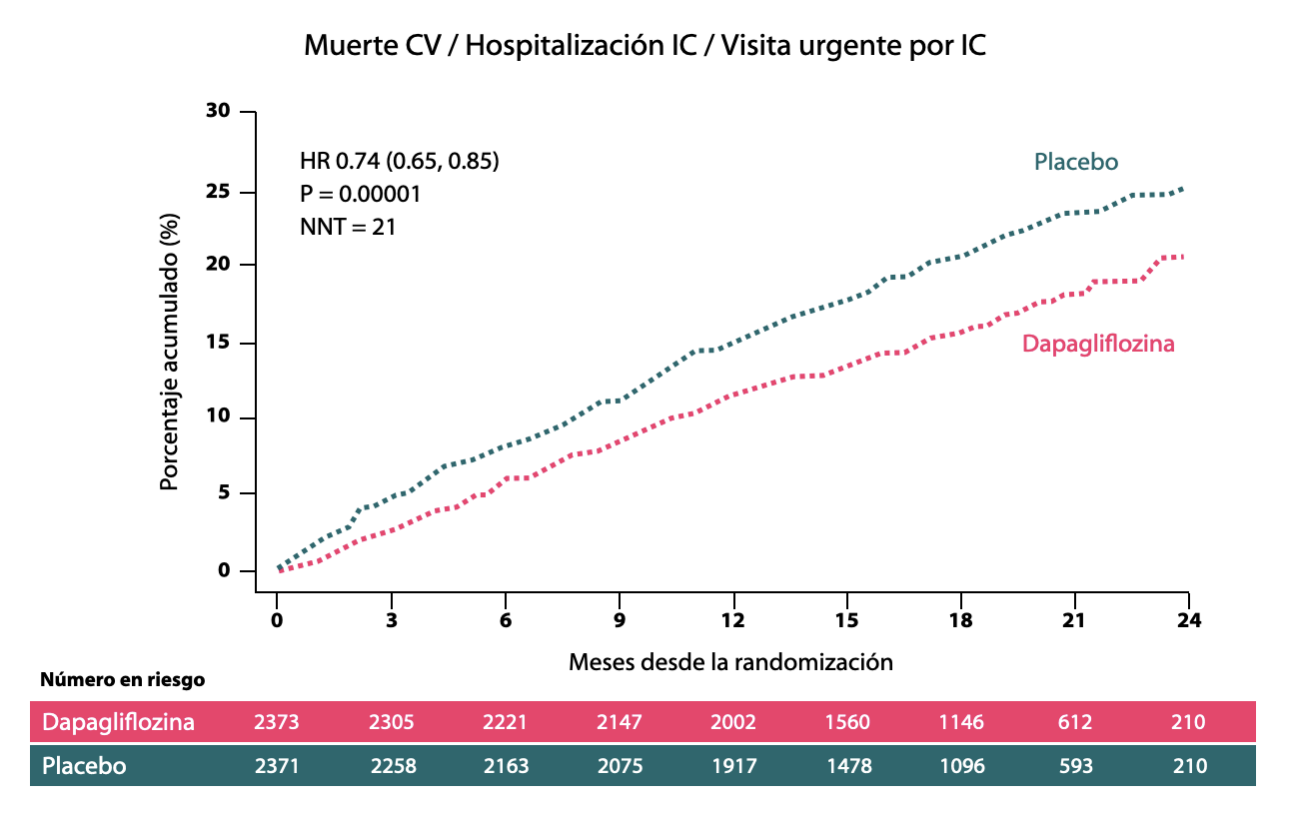
Resultados del estudio DAPA-HF. Adaptado de McMurray J. Dapaglifozin in patients with heart failure and reduced EF. N Eng J Med 2019. IC: Insuficiencia cardiaca; CV: Cardiovascular.

El estudio DAPA-HF reclutó 4,744 pacientes con IC CF II-IV y Fracción de Eyección menor a 40%, se aleatorizó para recibir dapaglifozina 10mg o placebo. El objetivo primario consistió en el punto combinado de muerte cardiovascular, hospitalización por insuficiencia cardiaca o una visita a emergencia por IC. El tiempo de seguimiento fue de tres años aproximadamente. Los resultados presentados en el congreso ESC 2019 en París muestran que dapaglifozina redujo el objetivo compuesto en un 26% (p<0.0001) (Figura 4) y mostró reducción en cada uno de los componentes individuales del objetivo primario. NNT 21 en el primer mes y otros beneficios como mejoría en la calidad de vida y 17% reducción en la mortalidad por todas las causas, con menos del 5% de suspensión y baja tasa de eventos adversos. La magnitud del beneficio fue independiente de la presencia de diabetes. Sin embargo, existen algunos puntos por resolver:

- ¿El beneficio será igual en pacientes con IC de mayor severidad? (En DAPA-HF 80% pacientes en Clase funcional II NYHA)
- Sólo el 10% de pacientes usó sacubitril/valsartán ¿será similar el beneficio y efectos adversos?
- ¿Cuál es el mecanismo de beneficio? En no diabéticos el efecto diurético parece ser irrelevante.
- ¿Hablaremos pronto de cuádruple bloqueo neurohumoral?
- ¿Lo iniciaremos durante la hospitalización?
- ¿En pacientes con ICFEp será el primer grupo farmacológico que disminuya la morbi-mortalidad cardiovascular?

Posiblemente, obtendremos respuestas en los siguientes años a partir de estudios que aún están en curso tanto con dapaglifozina (DELIVER) y empaglifozina (EMPEROR-Reduced y EMPEROR-Preserved).^60^^-^^61^

## Conclusiones

Diabetes Mellitus e Insuficiencia Cardiaca mantienen una estrecha relación fisiopatológica y peligrosa. El tratamiento estricto de cada una de estas patologías según la medicina basada en la evidencia disminuirá la posibilidad de la aparición de la otra. Lo cierto es que el manejo de la diabetes mellitus en el paciente con enfermedad cardiovascular e insuficiencia cardiaca va mas allá del control glucémico; destacando claramente los inhibidores de SGLT2. Además, los resultados del estudio DAPA-HF marcan una nueva era en el tratamiento de la IC.

## Financiación:

Ninguna
